# Puerto Rico Cancer Research Meeting: a venue designed by graduate students to strengthen Puerto Rico's scientific environment

**DOI:** 10.1091/mbc.E25-10-0481

**Published:** 2025-12-16

**Authors:** Ivette J. Suárez-Arroyo, Vicmag Cabrera-Rolón, Michelle M. Martínez-Montemayor

**Affiliations:** ^1^Universidad Central del Caribe-School of Medicine, Bayamón, Puerto Rico 00960-6032; Goucher College

## Abstract

Puerto Rico Cancer Research Meeting (PRCRM) was conceived and established by four graduate students in response to the urgent need for specialized research forums on an Island that is geographically limited. PRCRM emphasizes collaborative efforts between local and national institutions, encouraging exchange of ideas that often lead to the development of new grant applications and research initiatives, empowering local investigators. Defining features of PRCRM are its commitment to inclusivity, the four graduate students completed their doctoral degrees and hold leadership positions at UCC, PHSU, NIH, Memorial Sloan Kettering, and, despite numerous challenges faced, the meeting ensures Puerto Ricans are represented.

## Abstract (executive summary)

- Puerto Rico Cancer Research Meeting (PRCRM) was conceived and established by four graduate students in response to the urgent need for specialized research forums on an Island that is geographically limited.
- PRCRM emphasizes collaborative efforts between local and national institutions, encouraging the exchange of ideas that often lead to the development of new grant applications and research initiatives, empowering local investigators.
- Defining features of PRCRM are its commitment to inclusivity, the four graduate students completed their doctoral degrees and hold leadership positions at UCC, PHSU, NIH, Memorial Sloan Kettering, and, despite numerous challenges faced, the meeting ensures Puerto Ricans are represented.

## INTRODUCTION

The Puerto Rico Cancer Research Meeting (PRCRM) was established in 2014 in response to the urgent need for specialized forums in cancer research that facilitate cross-disciplinary exchanges among investigators, patient advocates, and cancer survivors. Researchers in Puerto Rico frequently face challenges related to geographical isolation, including limited access to peers, scientific seminars, and collaborative networks. These barriers reduce opportunities for knowledge exchange and formal and informal collaborations, which are critical for scientific innovation (Ralfs, 2024). By providing a dedicated meeting space, the PRCRM has become a pivotal platform to address these challenges and foster a transformative landscape within Puerto Rico's scientific community.

The inaugural PRCRM was conceived by four graduate students from two private medical schools: Universidad Central del Caribe (UCC) and Ponce Health Sciences University (PHSU). These students identified pressing challenges faced by Puerto Rican scientists in cancer research, including underrepresentation in leadership roles, limited access to funding and resources, and the absence of strong support networks.

The PRCRM strategically incorporates plenary speakers and, most importantly, local talk sessions, tailored to provide a platform for local investigators to disseminate cutting-edge cancer research. These sessions foster a more informed and interconnected scientific community, nurturing the Island's scientific foundation. Central to PRCRM's mission is the empowerment of voices that have traditionally faced barriers in accessing scientific platforms. The incorporation of short-talk sessions for students and postdoctoral researchers creates affordable opportunities for trainees to showcase their work, catalyzing their professional growth and bolstering representation within the biomedical workforce.

Furthermore, the PRCRM emphasizes collaborative efforts between local and national institutions, fostering an inspiring environment that encourages exploration of diverse perspectives and methodologies. Plenary and poster sessions promote active exchange of ideas, interactions, and long-term partnerships, often leading to the development of new grant applications and research initiatives. These interactions significantly amplify the impact of this initiative, fortifying Puerto Rico's scientific environment and empowering investigators to make meaningful contributions to cancer research at both national and global scales.

## MEETING STRUCTURE

The PRCRM is a one-day event held annually on the first Friday of October in Puerto Rico. Held in San Juan, Puerto Rico, with 129 participants, the fifth edition of the PRCRM gathered researchers from Cincinnati Children's Hospital, Vanderbilt University, University of Puerto Rico, representatives from the Puerto Rico Science, Technology, and Research Trust, a program officer from the NCI SBIR/STTR division, and industry partners like CytoImmune Therapeutics Puerto Rico. The fifth edition featured a dynamic program that included two plenary talks by invited national speakers, five presentations from local researchers and patient advocates, four short oral talks delivered by students and postdoctoral fellows, and a poster session showcasing diverse cancer research projects.

It is important to highlight that the 2017 edition was canceled following the devastation caused by Category 5 hurricanes, Irma and María, while the 2020–2022 editions were suspended due to the effects of a major earthquake that affected Puerto Rico and the SARS-Cov-2/COVID-19 pandemic.

## RESULTS AND HIGHLIGHTS OF THE PRCRM

**FIGURE 1:**
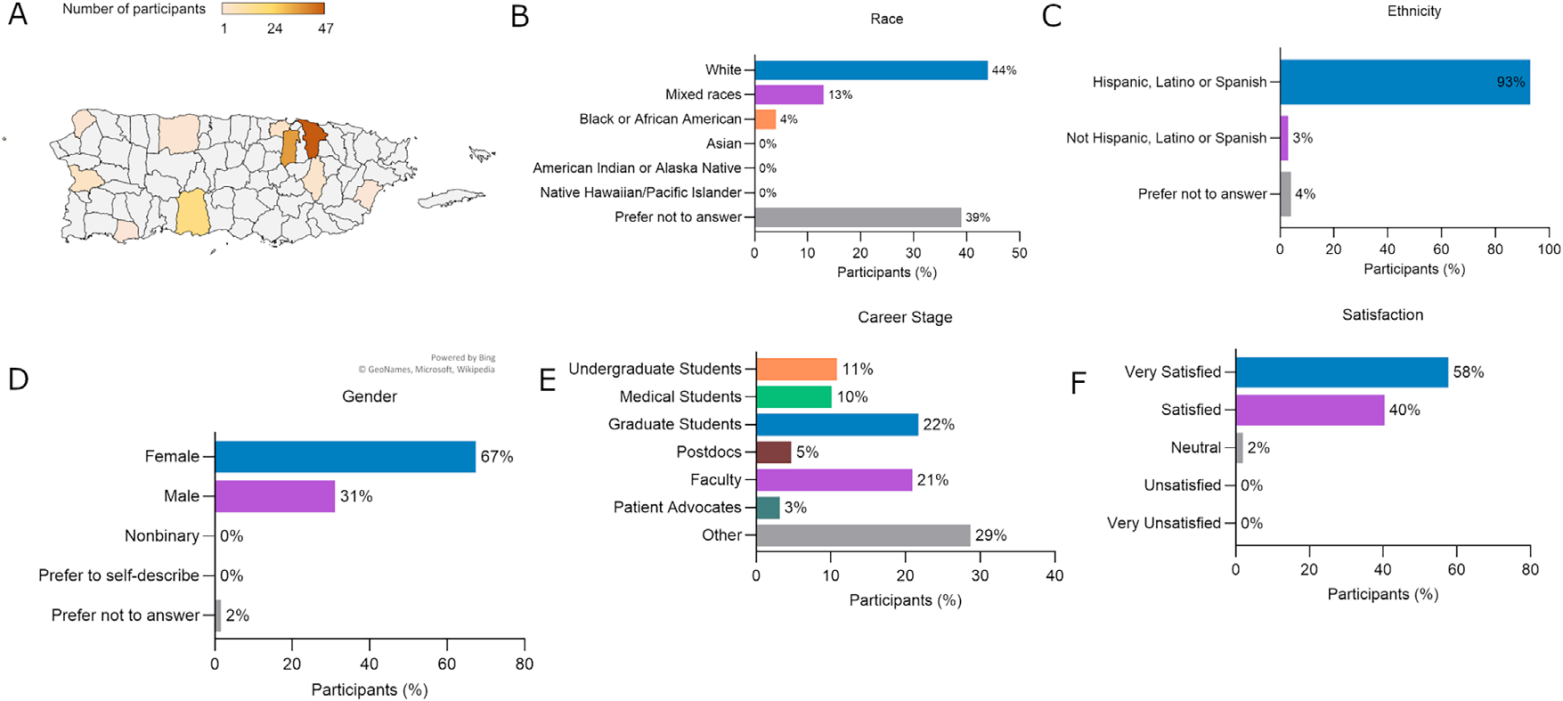
The fifth Puerto Rico Cancer Research Meeting participant profile. (A) Geographical distribution of the 2024 Puerto Rico Cancer Research Meeting participants from Puerto Rico (*n* = 118). (B) Race of participants (*n* = 128). (C) Ethnicity of participants (*n* = 129). (D) Gender of participants (*n* = 129). (E) Career stage of participants (*n* = 129). (F) Participants’ satisfaction experience (*n* = 52).

We implemented multiple approaches to assess participation and engagement among attendees. Over the years, PRCRM has drawn a diverse participant base from both Puerto Rico and the United States. In recent and previous editions, most local attendees were from the metropolitan and Southern regions of the island, where major research centers and medical schools, including UCC, are located (Figure 1A). Nationally, over 50% of speakers and participants were represented by institutions from the Eastern and Southern United States, an expected trend given Puerto Rico's geographic proximity to the East Coast, which facilitates strong and long-lasting research collaborations.

A defining feature of PRCRM is its commitment to inclusivity. The majority of participants identified their ethnicity as “Hispanic, Latino, or Spanish” (93%) and their gender as “Female” (67%), demographics that remain underrepresented in biomedical sciences (Figure 1, B–D). Analysis of participants’ career stages revealed that over 43% were undergraduate, medical, or graduate students, 21% held faculty positions, 5% were postdocs, and 3% were patient advocates. Attendees included in the “other” category (29%) included scientists in academic administrative roles (deans and presidents), directors or CEO of research of private entities, industry, program officers from the National Institutes of Health (NIH), or nonspecific roles (Figure 1E). Importantly, several trainees who presented posters or talks in recent and past editions are currently pursuing graduate or postdoctoral training or serving as faculty members at participant institutions such as UCC, MD Anderson Cancer Center, Wake Forest University, and New York University. A significant outcome is that the four original graduate student founders of the PRCRM have all earned their doctoral degrees and hold research or leadership positions at UCC, NIH, Memorial Sloan Kettering Cancer Center, and PHSU.

The 2024 edition, supported in part by an NIH/National Cancer Institute R13 Research Conference Grant, marked a major milestone in the event's evolution. We received 60 abstracts from researchers at various academic levels. The top four abstracts were selected for oral presentations, and the remaining were showcased in the poster session. We provided awards to the top three poster presenters, as determined by evaluation from faculty attendees, senior doctoral students, and patients.

One of the meeting's most inspiring outcomes was the oral presentation delivered by a young Puerto Rican investigator who completed her PhD at UCC and who subsequently secured an American Cancer Society-funded postdoctoral fellowship at New York University (NYU). She returned to the Island with the vision of establishing herself as an independent researcher. Her participation in PRCRM led to an interview for a faculty position at PHSU, where she was successfully recruited, exemplifying how PRCRM serves as a catalyst for scientific careers on the island. The meeting also fostered numerous collaborations among local researchers, as well as between Puerto Rican and US-based investigators.

Post-event evaluations reflected the meeting's success. Respondents were “very satisfied” or “satisfied” (98%) with the activity (Figure 1F). Some expressed that the PRCRM helped them expand their knowledge of cancer research, met their expectations, encouraged collaboration, and was an event they would recommend to others.

## CONCLUSION

Despite the numerous challenges faced, the PRCRM continues to thrive as an important forum for local cancer researchers. Its success demonstrates a strong commitment to advancing cancer research in Puerto Rico. Looking forward, the PRCRM will continue to create opportunities for collaboration, visibility, and career advancement, ensuring that Puerto Rican investigators remain integral contributors to the national and global cancer research.

**Figure:**
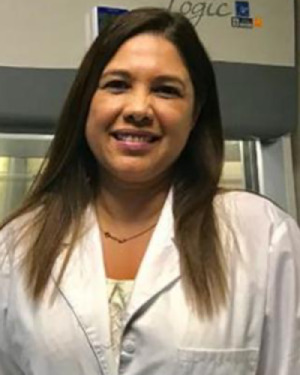


**Ivette J. Suárez-Arroyo** I am currently an Associate Professor in the Department of Biochemistry at Universidad Central del Caribe (UCC), Puerto Rico. I earned my Ph.D. in Cellular and Molecular Biology from UCC and completed postdoctoral training in cancer biology. My research focuses on the investigation of molecular mechanisms of breast cancer progression, with particular emphasis on signaling pathways and their roles in chemotherapy resistance. I am deeply committed to mentoring emerging scientists and enhancing research capacity in Puerto Rico. I am one of the founding members and current co-leader of the Puerto Rico Cancer Research Meeting (PRCRM), a trainee-driven platform designed to foster visibility, collaboration, and leadership among local and national cancer researchers.

**Figure:**
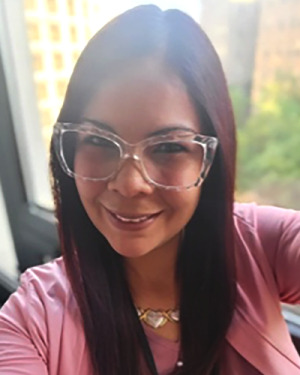


**Vicmag Cabrera-Rolón** I am currently the sponsored program officer at the Universidad Central del Caribe (UCC), Puerto Rico. I earned a bachelor's degree in office systems from the University of Puerto Rico. I have been part of the Alliance for Clinical and Translational Research (Alliance) Tracking and Evaluation Core since 2020 and have extensive expertise in quantitative and qualitative data management, project activities and management, subcontract agreements, research projects coordination, submission of grant proposals, and reports. As part of my responsibilities, I maintain the UCC research products databases, including grant submissions, grants approved, active research projects, manuscripts submitted, publications, and presentations, among other important duties for all UCC researchers.

**Figure:**
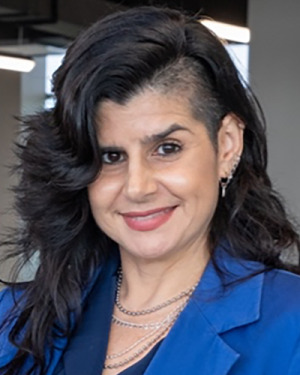


**Michelle M. Martínez-Montemayor** I am a Native Puerto Rican and a first-generation graduate student. I completed my undergraduate studies and master's degree at the University of Puerto Rico (UPR Bayamón, Cayey, and Mayagüez Campus, respectively). Then I earned my PhD in animal science at Michigan State University and completed two postdoctoral experiences in molecular and cellular cognition, and cancer biology at UPR Rio Piedras, and Universidad Central del Caribe School of Medicine (UCC), respectively. I am currently a Professor in the Department of Biochemistry at UCC, where I study natural product-derived therapies for aggressive breast cancers. Recently co-founded and presided over Dynamiko Pharmaceutics, a startup with the mission of developing selective anti-cancer therapies. I have devoted my life to training and mentoring the next generation of scientists. I currently serve as a member of the American Society for Cell Biology (ASCB) Maximizing Access to Cell Biology for PEERS Committee (MAC), and co-lead the Puerto Rico Cancer Research Meeting (PRCRM).

## Abbreviations used:

NIH: National Institutes of Health
NCI: National Cancer Institute
NYU: New York University
PHSU: Ponce Health Sciences University
PRCRM: Puerto Rico Cancer Research Meeting
SBIR: Small Business Innovation Research
STTR: Small Business Technology Transfer
UCC: Universidad Central del Caribe School of Medicine.
